# Percutaneous Orthotopic Transcatheter Tricuspid Valve Replacement for Native Valve Destruction After Endocarditis

**DOI:** 10.1016/j.jaccas.2025.105280

**Published:** 2025-10-01

**Authors:** Andrea Ruberti, Laura Sanchis, Andrea Arenas-Loriente, Blanca Domenech-Ximenos, Eduardo Flores-Umanzor, Ander Regueiro, Marta Farrero, Marta Sitges, Xavier Freixa, Omar Abdul-Jawad Altisent

**Affiliations:** a Cardiovascular institute, Hospital Clínic, Barcelona, Spain; b Institut d'Investigacions Biomèdiques Agustí Pi i Sunyer (IDIBAPS), Barcelona, Spain; c Universitat de Barcelona, Barcelona, Spain; d Centre de diagnòstic per la Imatge, Hospital Clinic, Barcelona, Spain; e CIBER Cardiovascular, Instituto Carlos III, MADRID, Spain

**Keywords:** endocarditis, LuX-Valve, tricuspid regurgitation, TTVR

## Abstract

**Background:**

Transcatheter tricuspid valve replacement (TTVR) is emerging as an option for treating severe tricuspid regurgitation (TR) in patients at high surgical risk.

**Case Summary:**

A 59-year-old man with torrential TR and right heart failure following *Staphylococcus aureus* tricuspid valve infective endocarditis and extensive leaflet destruction was successfully treated with orthotopic TTVR using the LuX-Valve Plus system (Jenscare), resulting in significant clinical improvement and favorable right ventricular remodeling.

**Discussion:**

The LuX-Valve Plus, delivered via transjugular access, enables optimal coaxial alignment during deployment. Its unique features—leaflet graspers, a septal anchoring mechanism, and a flexible frame—provide stability and preserve right ventricular function, making it suitable for complex tricuspid anatomies.

**Take-Home Messages:**

Orthotopic TTVR with the LuX-Valve Plus offers a viable option for patients with severe TR and extensive leaflet destruction, previously considered ineligible for surgical or transcatheter repair. Multimodality imaging is essential for procedural planning and optimal outcomes.

## Graphical Abstract

**Figure:**
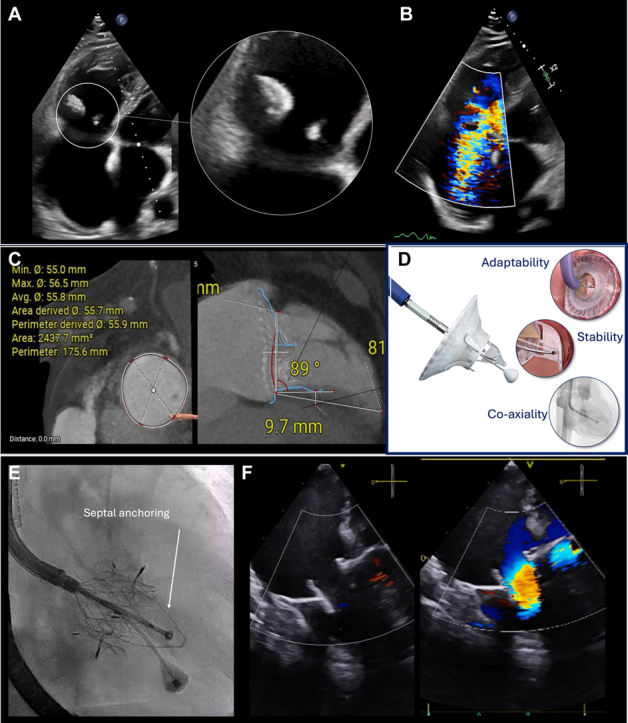

## History of Presentation and Past Medical History

A 59-year-old man was initially admitted with *methicillin-susceptible Staphylococcus aureus* infective endocarditis (IE) involving the tricuspid valve (TV), leading to extensive valve damage and septicemia. The patient declined open-heart surgery and was instead managed conservatively with prolonged intravenous antibiotic therapy during an extended intensive care unit stay. He responded well to medical management and was eventually discharged.

**Take-Home Messages**

• Orthotopic TTVR with the LuX-Valve Plus offers a viable option for patients with severe TR and extensive leaflet destruction, previously considered ineligible for surgical or transcatheter repair.
• Careful multimodality imaging is essential to guide procedural planning and optimize outcomes in anatomically complex TV interventions.

Six months later, the patient presented signs of persistent right heart failure and poor functional status [NYHA functional class III] despite being on high-dose diuretics (furosemide, 120 mg/d). On physical examinations, he was afebrile with normal arterial pressure, a heart rate of 100 beats/min, systolic murmur in the tricuspid area, jugular engorgement, pulsatile hepatomegaly, and mild edema in the lower extremities.

## Investigations

**Figure 1:**
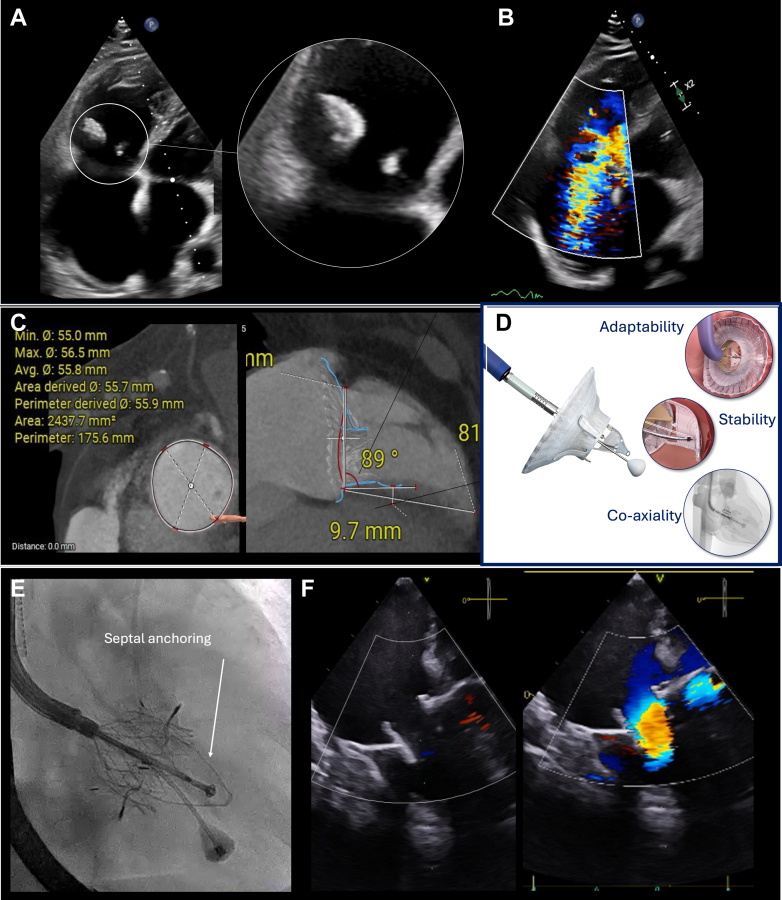
Baseline Echocardiography and CT Scan, Main Lux-Valve Plus Features, Fluroscopic View During Implantation and Final Echocardigraphic Result (A and B) Baseline transthoracic echocardiography (TTE) showing aseptic vegetations (left, white circle) and torrential tricuspid regurgitation (TR) (right). (C) Computed tomography (CT) scan assessing anatomical suitability for transcatheter tricuspid valve replacement (TTVR). (D) The LuX-Valve plus system (Jenscare) highlighting key features: coaxial alignment via jugular approach, stability through septal anchoring, and adaptability with a flexible design and large atrial skirt. (E) Fluoroscopic view demonstrating LuX-Valve system (Jenscare) deployment stabilized by the septal anchor (white arrow). (F) X-plane transesophageal echocardiography (TEE) confirming the final successful result.

Echocardiography revealed torrential TR—due to anterior and septal leaflet tethering—2 large vegetations (2 cm each), and severe right ventricular (RV) dysfunction (tricuspid annular plane systolic excursion 13 mm, RV ejection fraction 22%) (Figures 1A and 1B, Videos 1 and 2). The electrocardiogram showed atrial fibrillation with right bundle branch block. Repeated blood cultures remained negative, laboratory tests showed no signs of ongoing infection, and a positron emission tomography scan ruled out any hypermetabolic activity in the tricuspid area. Right heart catheterization confirmed moderate pulmonary hypertension (mean pulmonary artery pressure 32 mm Hg, mean pulmonary capillary wedge pressure 25 mm Hg, pulmonary vascular resistance 2.11 WU). The 6-minute walk test distance was 250 m, and the Kansas City Cardiomyopathy Questionnaire score was 48.

## Management

The patient was considered to have sequelae of prior TV endocarditis, presenting with 2 large aseptic vegetations resulting in torrential TR. There were no signs of active infection; therefore, suppressive antibiotic therapy was not indicated. Given the high surgical risk (TRI-Score predicted in-hospital mortality of 22%) and the patient's persistent refusal of open-heart surgery, a percutaneous treatment approach was proposed. Due to the extensive destruction of the leaflets, percutaneous TV repair was not feasible. A computed tomography scan (Figure 1C) confirmed favorable anatomy for percutaneous intervention. A 30- to 60-mm LuX-Valve Plus system (Jenscare) was implanted via a 34F right transjugular access (Figures 1D and 1E). The procedure was successful, with no residual TR, a mean transvalvular gradient of 2 mm Hg, and mPAP of 25 mm Hg (Video 3, Video 4, Video 5). The patient was discharged 3 days after the procedure without complications.

## Outcome and Follow-Up

**Figure 2:**
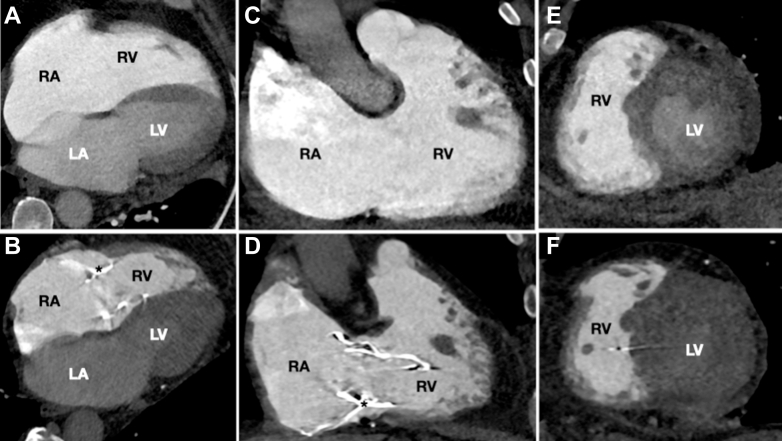
Cardiac CT Scan Before and After the Procedure Cardiac CT scans at baseline (A, C, and E) and at 1-year follow-up (B, D, and F) after orthotopic transcatheter tricuspid valve replacement (TTVR) using the Lux-Valve Plus system, demonstrating significant RV and RA reverse remodeling. The maximal RV basal diameter decreased from 54 mm to 41 mm, and RA area was reduced from 50 to 32 cm^2^. (A and B) Four-chamber views; (C and D) two-chamber views; and (E and F) transverse views. LA = left atrium; LV = left ventricle; RA= right atrium; RV = right ventricle.

At 6 months following the TTVR, the patient showed marked clinical and functional improvement, including a NYHA functional class I, a 6-minute walk test of 420 m, and a Kansas City Cardiomyopathy Questionnaire score of 91. Imaging confirmed complete resolution of TR, improvement in RV function (tricuspid annular plane systolic excursion 18 mm, RV ejection fraction 26%), and favorable RV remodeling (Video 6). These gains were sustained at the 1-year follow-up (Figure 2).

## Discussion

Right-sided IE, although relatively uncommon, poses significant therapeutic challenges, particularly when complicated by significant TV destruction and RV dysfunction. Most associated with intravenous drug use or cardiac implantable electronic devices, and severe cases often progress to persistent right heart failure and sepsis, with limited treatment options.^1^ While open-heart surgery remains the standard for advanced IE, many patients are poor surgical candidates due to high perioperative risk. In this high-risk population, less invasive alternatives are critically needed.

Transcatheter tricuspid interventions, such as edge-to-edge repair and orthotopic TTVR, are evolving treatment options. However, their utility is often constrained by complex anatomy and extensive leaflet destruction, a common sequela of IE.^2^ Tricuspid edge-to-edge repair, in particular, depends on the presence of adequate leaflet length, structural integrity, and mobility to achieve effective coaptation. In the present case, these prerequisites were not met: The septal leaflet was extensively damaged and retracted, and the anterior leaflet was partially destroyed and burdened by a large vegetation, and only a severely tethered posterior leaflet remnant remained. This made successful leaflet approximation technically unfeasible. Orthotopic TTVR, while a more recent acquisition to the percutaneous armamentarium, is similarly challenged by anatomical limitations. The current available device, the Evoque system (Edwards Lifesciences), relies on the integrity of all native leaflets to ensure anchoring, which constrains its utility in cases of profound leaflet destruction.^3^^,^^4^

**Figure 3:**
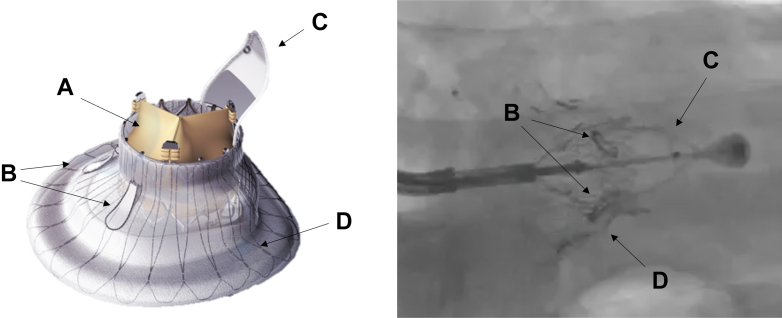
LuxValve Plus Anchoring System (A) Prosthetic valve biologic leaflets; (B) graspers; (C) interventricular septal anchor; (D) valve disk.

The LuX-Valve Plus system incorporates several design advantages tailored to these challenging anatomies.^5^ Unlike other orthotopic TTVR systems that rely primarily on radial force for fixation, the LuX-Valve Plus system utilizes a dual-element anchoring strategy. This includes 2 leaflet graspers, typically securing the anterior, or both the anterior and posterior leaflet, and a dedicated interventricular septal anchor. This configuration enables stable implantation that is relatively independent of leaflet integrity, offering enhanced procedural security even in cases of severe leaflet destruction (Figure 3, Video 3). Despite these advantages, certain limitations remain. Implantation may be particularly challenging in cases of complete anterior leaflet loss, as the leaflet graspers may lack sufficient tissue for secure engagement. In addition, active IE is a contraindication due to the risk of prosthetic infection and poor tissue quality for anchoring.

Another notable feature of the Lux-Valve Plus is its transjugular access route, which facilitates more coaxial alignment with the tricuspid annulus, simplifying the procedure and reducing imaging requirements compared with other devices. In addition, the device's flexible and size-adaptable frame accommodates a wide range of anatomical configurations while mitigating the risk of postprocedural RV stunning—a common concern in TTVR procedures.^2^ Collectively, these characteristics make the LuX-Valve Plus system particularly advantageous for high-risk patients with complex, nonrepairable TV anatomy. Nonetheless, the importance of detailed preprocedural planning and intraprocedural guidance using multimodality imaging is essential to ensure successful and safe deployment.

Orthotopic TTVR after tricuspid IE has been reported in patients with prior surgical TV surgery who subsequently developed recurrent valve dysfunction. However, to the best of our knowledge, this is the first reported case of severe TR following IE of native TV treated successfully with an orthotopic TTVR strategy. This case highlights the potential of LuX-Valve Plus system to expand percutaneous treatment options in previously untreatable patients, representing a significant step forward in structural interventions.

Future research should focus on refining risk-prediction models to improve procedural planning and long-term prognostication. Further investigation is needed to explore TTVR strategy in challenging scenarios and to evaluate long-term RV remodeling, with the goal to optimize post-TTVR management and patient selection. A personalized approach that integrates TR pathophysiology, RV remodeling, and therapeutic response may enhance outcomes in TTVR recipients. Ongoing studies will further clarify the mid- and long-term outcomes will define the role of the LuX-Valve Plus system in patients with severe TR (TRINITY-US trial, NCT06568003).

## Conclusions

Orthotopic TTVR may be an effective treatment option for severe TR, even in anatomically complex cases such as those following native valve IE. Meticulous preprocedural planning using multimodality imaging is essential to ensure procedural success and optimal patient outcomes.

## Funding Support and Author Disclosures

Dr Altisent is a proctor of Products & Features and Abbott. Dr Freixa is a proctor for Abbott and LifeTech Science. Dr Sitges is a consultant and speaker for General Electric, Medtronic, Edwards Lifesciences, and Abbott. Dr Sanchis is a proctor for Abbott and Edwards Lifesciences and a speaker for GE Healthcare, Abbott, and Edwards Lifesciences. All other authors have reported that they have no relationships relevant to the contents of this paper to disclose.

## Appendix

Video 1 Four-Chamber-View Transthoracic Echocardiography (TTE), 2D and Color Doppler, Tricuspid Valve Endocarditis With Torrential Tricuspid Regurgitation

Video 2 Right Ventricle Two-Chamber View, 2D, Color Doppler, and 3D Transesophageal Echocardiography (TEE), Tricuspid Valve Endocarditis With Torrential Tricuspid Regurgitation

Video 3 Fluoroscopy, Lux-Valve Plus (Jenscare) Deployment

Video 4 Intraprocedural Echocardiography (TEE), Lux-Valve Plus (Jenscare) Deployment

Video 5 Intraprocedural Echocardiography (TEE), Final Result After Transcatheter Tricuspid Valve Replacement With Lux-Valve Plus (Jenscare)

Video 6 Four-Chamber-View Transthoracic Echocardiography (TTE) at 6 Months, Favorable Right Ventricular Remodeling
